# First-degree relationships and genotyping errors deciphered by a high-density SNP array in a Duroc × Iberian pig cross

**DOI:** 10.1186/s12863-022-01025-1

**Published:** 2022-02-17

**Authors:** L. Gomez-Raya, E. Gómez Izquierdo, E. de Mercado de la Peña, F. Garcia-Ruiz, W.M. Rauw

**Affiliations:** 1 Departamento de Mejora Genética Animal, Instituto Nacional de Investigación y Tecnología Agraria y Alimentaria (INIA-CSIC), Ctra. de La Coruña km 7.5, 28040 Madrid, Spain; 2 Centro de Pruebas de Porcino, Instituto Tecnológico Agrario Junta de Castilla y León (ITACyL), Ctra Riaza-Toro S/N, 40353 Hontalbilla, Spain; 3 Departamento de Reproducción Animal, Instituto Nacional de Investigación y Tecnología Agraria y Alimentaria (INIA-CSIC), Avda. Puerta de Hierro s/n, 28040 Madrid, Spain

**Keywords:** Single Nucleotide Polymorphism, Genotyping errors, Paternity test, HD SNP array, Pig

## Abstract

**Background:**

Two individuals with a first-degree relationship share about 50 percent of their alleles. Parent–offspring relationships cannot be homozygous for alternative alleles (genetic exclusion).

**Methods:**

Applying the concept of genetic exclusion to HD arrays typed in animals for experimental purposes or genomic selection allows estimation of the rate of rejection of first-degree relationships as the rate at which two individuals typed for a large number of Single Nucleotide Polymorphisms (SNPs) do not share at least one allele. An Expectation–Maximization algorithm is applied to estimate parentage. In addition, genotyping errors are estimated in true parent–offspring relationships. Samples from nine candidate Duroc sires and 55 Iberian dams producing 214 Duroc × Iberian barrows were typed for the HD porcine Affymetrix array.

**Results:**

We were able to establish paternity and maternity of 75 and 85 piglets, respectively. Rate of rejection in true parent–offspring relationships was estimated as 0.000735. This is a lower bound of the genotyping error since rate of rejection depends on allele frequencies. After accounting for allele frequencies, our estimate of the genotyping error is 0.6%. A total of 7,744 SNPs were rejected in five or more true parent–offspring relationships facilitating identification of “problematic” SNPs with inconsistent inheritance in multiple parent–offspring relationships.

**Conclusions:**

This study shows that animal experiments and routine genotyping in genomic selection allow to establish or to verify first-degree relationships as well as to estimate genotyping errors for each batch of animals or experiment.

**Supplementary Information:**

The online version contains supplementary material available at 10.1186/s12863-022-01025-1.

## Background

Next Generation Sequencing (NGS), a term used for massive parallel sequencing of several hundred thousand to millions of DNA fragments simultaneously, has enabled massive discovery of novel single nucleotide polymorphism (SNP) genetic markers [1]. Today, high density (HD) SNP arrays allow interrogating a genome for hundreds of thousands of SNPs at a time. Subsequently, HD SNP arrays have been used to interrogate human, animal and plant genomes for SNPs associated to disease and production traits [2, 3].

In livestock production, genotyped individuals in a population are often much more related than in human research. In most cases, individuals with first-degree relationships, i.e., parent–offspring or full-sib relationships, are part of the same selection candidates in genomic selection or crossbreeding programs. For optimum contribution selection (OCS), restricting the relationship between selected parents is crucial to restrict inbreeding in the progeny and maximize genetic gain [4]. Therefore, for genomic breeding value estimation, the genetic relationships between individuals based on markers densely distributed across the genome need to be accurately established across individuals [5]. In practice, this requirement may be hindered by instances of incorrect labelling or registration of individuals with their DNA samples. In addition, virtually every genetic study includes genotyping errors, quantified by the Quality scores probability of error provided by genomics companies, resulting from calling algorithms that misidentify and misclassify the individual’s genotype [6]. It is likely that genotyping errors vary between experiments due to variation in DNA quality or lab sample handling. Genotyping errors are usually not reported in scientific studies.

The objective of this study is to investigate the rate of rejection of first-degree relationships of individuals in a population using an HD SNP array. The second objective of this study is to demonstrate the use of HD SNP arrays in detecting genotyping errors after parent–offspring relationships are resolved. The third objective is to identify SNPs that repeatedly result in genotyping errors in multiple true parent–offspring relationships.

## Methods

### Genome-wide rate of rejection of first-degree relationships among all individuals

A first-degree relative is an individual who shares approximately 50 percent of their alleles with a particular other individual. There are two first-degree relationships: parent-offspring, and full-sibs. Under strict Mendelian inheritance, individuals with a first-degree parent–offspring relationship cannot be homozygous for alternative alleles at a given locus. For example, an individual offspring cannot be homozygous CC at a locus, when a parent is GG at the same locus. Applying this concept to a population in which individuals are genotyped with an HD SNP array allows the rejection of parent–offspring first-degree relationships based on this rule. Full-sibs, on the other hand, can be homozygous for alternative alleles when both parents are heterozygous, but this probability is small, at 0.125.

We define genome-wide rate of rejection of first-degree relationships as the rate at which two individuals genotyped for a large number of SNPs have alternatives alleles, i.e., the number of markers for which two individuals are homozygous for alternative alleles divided by the total number of SNPs genotyped with an HD SNP array. The rate of rejection of a first-degree relationship between two individuals *i* and *j* is thus defined as:

1 $${\tau }_{ij}=\frac{\sum_{i=1}^{{N}_{snp}}{s}_{i}}{{N}_{snp}}$$

where *N*_snp_ is the total number of SNPs in the array; for each SNP, ${s}_{i}$ is a dummy variable with value 1 if the first-degree relationship is rejected for the *i-th* marker (i.e., individuals are homozygous for alternative alleles at that locus); it is 0 otherwise.

Estimates of *τ*_*ij*_ corresponding to each relationship can be used to detect or confirm first-degree relationships and genotyping errors. In Eq. (1), *τ*_*ij*_ = 0 implies that individuals *i* and *j* are not homozygous for alternative alleles at any of the SNP markers in the array, therefore, they have a parent–offspring first-degree relationship; *τ*_*ij*_ > 0 implies there is not a parent–offspring first-degree relationship. However, full-sibs with a true first-degree relationship can be homozygous for alternative alleles when both parents are heterozygous, therefore *τ*_*ij*_ > 0. In addition, when genotyping errors occur, individuals *i* and *j* may appear to be homozygous for alternative alleles at some of the SNP markers in the HD SNP array when in reality they are not, such that *τ*_*ij*_ ≈ 0. In that case, it would mean a rejection of a true parent–offspring first-degree relationship. Because genotyping errors do occur, a method needs to be developed that can distinguish values of *τ*_*ij*_ ≈ 0 that result from genotyping errors from values of τ_ij_ > 0 resulting from a true rejection of a parent–offspring first-degree relationship.

Values of τ_ij_ can be calculated for all possible relationships between all pairs of individuals in the population; in a population with *ni* individuals, the total number of relationships is (*ni*^2^ – *ni*)/2. The binomial density models the probability of each outcome according to:

$$p\left(\tau \right)=\left(\begin{array}{c}{N}_{snp}\\ \delta \end{array}\right){\left(\tau \right)}^{\delta }{\left(1-\tau \right)}^{{N}_{snp}-\delta }$$

where *p(τ)* is the probability of the rate of rejection,* τ*, at the number of SNPs rejecting first-degree relationships, $\delta =\sum_{i=1}^{{N}_{snp}}{s}_{i}$.

### Expectation–Maximization to estimate first-degree relationships

Given the distribution of the rate of rejection of first-degree relationships in a population, *τ*, when *τ*_*ij*_ > 0, mixing of several distinct distributions can be identified: one corresponding to binomial probabilities *τ*_*g*_ resulting from genotyping errors, and others corresponding to binomial probabilities τ_r_ resulting from rejections of first-degree relationships. Here, the rate of genotyping errors is a lower bound of the true rate of genotyping errors, since genotyping errors are only identified in SNP markers for which the individuals are homozygous for alternative alleles; genotyping errors in individuals that do not lead to a rejection of a first-degree relationship will remain undetected. In the dataset it is assumed that estimates of rates of rejection following Eq. (1) when *τ*_*ij*_ > 0 should belong either to the distribution of *τ*_*g*_ or to the distribution of *τ*_*r*_, i.e., a true first-degree relationship is rejected because of genotyping errors (the values are close to zero, *τ*_*ij*_ ≈ 0) *vs*. a first-degree relationship is rejected because it does not exist (the values are farther away from zero, *τ*_ij_ > 0). In order to determine to which of the two distributions a relationship belongs, the dataset has to be first subdivided into known relationship groups. In our example, we consider a crossbreeding experiment with candidate sires, dams and offspring. The data can now be subdivided into the following relationship groups: dam-offspring, sire-offspring, offspring-offspring, dam-dam, sire-sire, and sire-dam. First, we are interested in identifying dam-offspring, or sire-offspring relationships to establish maternities and paternities of our experimental crossbred population. A given pair of individuals from the dam-offspring or the sire-offspring group has a probability γ of belonging to the distribution of *τ*_*g*_ of true parent–offspring relationships. The likelihood function of the *i-th* relationship is:

2 $${L}_{i}\left(\gamma ,{\tau }_{g},{\tau }_{r}\right)\begin{array}{l}=\gamma {L}_{i}\left({\tau }_{g}\right)+ {\left(1-\gamma \right)L}_{i}\left({\tau }_{r}\right) \\ =\gamma \left[\left(\begin{array}{c}{N}_{snp}\\ {\delta }_{i}\end{array}\right){\left({\tau }_{g}\right)}^{{\delta }_{i}}{\left(1-{\tau }_{g}\right)}^{{N}_{snp}-{\delta }_{i}}\right]+(1-\gamma )\left[\left(\begin{array}{c}{N}_{snp}\\ {\delta }_{i}\end{array}\right){\left({\tau }_{r}\right)}^{{\delta }_{i}}{\left(1-{\tau }_{r}\right)}^{{N}_{snp}-{\delta }_{i}}\right]\end{array}$$

where ${\delta }_{i}$ is the number of SNPs rejecting the first-degree relationship. The joint likelihood function for all the relationships, *nr*, between all pairs of individuals within the dam-offspring or within the sire-offspring group is:

3 $$L\left(\gamma,\tau_g,\tau_r\right)={\textstyle\prod_{i=1}^{nr}}L_i$$

This likelihood has three unknowns: *γ*, *τ*_*g*_, and *τ*_*r*_. Maximizing this equation is not straightforward because Eq. (2) has an addition term, which makes using logarithms impractical. We can solve Eq. (3) by applying an Expectation–Maximization algorithm. This method requires starting values for *τ*_*g*_ and *τ*_*r*_. The expectation for the *i-th* relationship is:

4 $$\begin{array}{c}E\left[{t}_{g,i}\right]=\frac{{L}_{i}\left({\tau }_{g}\right)}{{L}_{i}\left({\tau }_{g}\right)+{L}_{i}\left({\tau }_{r}\right)}{\delta }_{i},\\ E\left[{t}_{r,i}\right]=\frac{{L}_{i}\left({\tau }_{r}\right)}{{L}_{i}\left({\tau }_{g}\right)+{L}_{i}\left({\tau }_{r}\right)}{\delta }_{i},\end{array}$$

The binomial probabilities are extremely small when the total number of SNPs is very large, therefore, it is convenient to manipulate these equations to:

$$\begin{array}{c}E\left[{t}_{g,i}\right]=\frac{1}{1+\frac{{L}_{i}\left({\tau }_{r}\right)}{{L}_{i}\left({\tau }_{g}\right)}}{\delta }_{i}\\ = \frac{1}{1+{e}^{(\mathrm{ln}{L}_{i}\left({\tau }_{r}\right)-\mathrm{ln}{L}_{i}\left({\tau }_{g}\right))}}{\delta }_{i}\\ \begin{array}{c}E\left[{t}_{r,i}\right]=\frac{1}{1+\frac{{L}_{i}\left({\tau }_{g}\right)}{{L}_{i}\left({\tau }_{r}\right)}}{\delta }_{i}\\ = \frac{1}{1+{e}^{(-\mathrm{ln}{L}_{i}\left({\tau }_{r}\right)+\mathrm{ln}{L}_{i}\left({\tau }_{g}\right))}}{\delta }_{i}\end{array}\end{array}$$

The maximization step is:

$$\begin{array}{c}{\tau }_{g}^{^{\prime}}=\frac{\sum_{i=1}^{nr}E\left[{t}_{g,i}\right]\frac{{\delta }_{i}}{{N}_{snp}}}{\sum_{i=1}^{nr}E\left[{t}_{g,i}\right]}\\ {\tau }_{r}^{^{\prime}}=\frac{\sum_{i=1}^{nr}E\left[{t}_{r,i}\right]\frac{{\delta }_{i}}{{N}_{snp}}}{\sum_{i=1}^{nr}E\left[{t}_{r,i}\right]}\end{array}$$

Parameter *γ*, i.e., the probability that *τ*_*ij*_ belongs to the distribution of *τ*_g_ of true parent–offspring relationships is estimated following:

$${\gamma }^{^{\prime}}=\frac{\sum_{i=1}^{nr}E\left[{t}_{g,i}\right]}{nr}$$

The process is iterative and parameters *τ*'_*g*_ and *τ'*_*r*_ estimated in one iteration are used for the next iteration as *τ*_*g*_ and *τ*_*r*_, respectively. Once convergence is reached, the *i-th* parent–offspring relationships are assigned as true when

$$\frac{{L}_{i}\left({\tau }_{g}\right)}{{L}_{i}\left({\tau }_{g}\right)+{L}_{i}\left({\tau }_{r}\right)}>0.5.$$

Once true parent–offspring relationships are identified, a lower bound of the estimate of the genotyping error is *τ*_*g*_. When this method is applied to the offspring-offspring relationship group, also full-sibs can be detected. In that case, the procedure does not attempt to estimate genotyping errors but will assign relationships according to two distributions with binomial parameter τ: one distribution with full-sibs and another distribution with any other relationship. The EM follows the same steps as for parent–offspring relationships as described above. 

### *Dataset of Duroc* × *Iberian Pigs*

The animal material from this study came from an experiment investigating production parameters in a commercial Duroc × Iberian pig cross [7]. In Spain, purebred Iberian pig meat (in particular dry-cured products) from pigs kept extensively in a production system called ‘montanera’ where they roam the Mediterranean forest and eat acorns is the most valuable meat product [8]. However, because of limited land availability and low production levels, Iberian pigs are regularly crossed with Duroc producing either 50% or 75% Iberian fattening pigs. In 2019, 50% crossbred Iberian pigs constituted 80% of the total Spanish Iberian pig production; 72% of those constituted Duroc × Iberian pigs fed intensively on concentrate [9]. The dataset consisted of nine candidate Duroc sires, 55 Iberian dams, and 214 Duroc × Iberian barrows. The true pedigree was unknown.

### Genotyping with the Porcine Affymetrix HD array

A total of 288 samples were genotyped with the HD porcine Affymetrix array (658,692 SNPs). One of the samples failed. Best Practices Workflow was applied with the following conditions in the Axiom Analysis Suite version 5.1.1.1: DQC: ≥ 0.82; QC call rate: ≥ 97; Average call rate for passing samples: ≥ 98.5; Percent of passing samples: ≥ 95. Of the 287 samples only 281 passed the QC-Call rate. There were 603,809 SNPs that passed the condition for the call rate. From those, only 546,220 autosomal SNPs mapped to SScroffa v11.1 were used for estimating rate of rejection of first-degree relationships. The genotyping was carried out at Centro Nacional de Genotipado (CeGen) at the University of Santiago de Compostela, Spain.

### Estimation of paternities, maternities and genotyping errors

The methods to estimate paternities, maternities, genotyping errors were as described in the Methods section. Source code in R language (http://www.r-project.org/) is provided as an additional file 1.

## Results

### Estimation and distribution of the rate of rejection of first-degree relationships

**Fig. 1:**
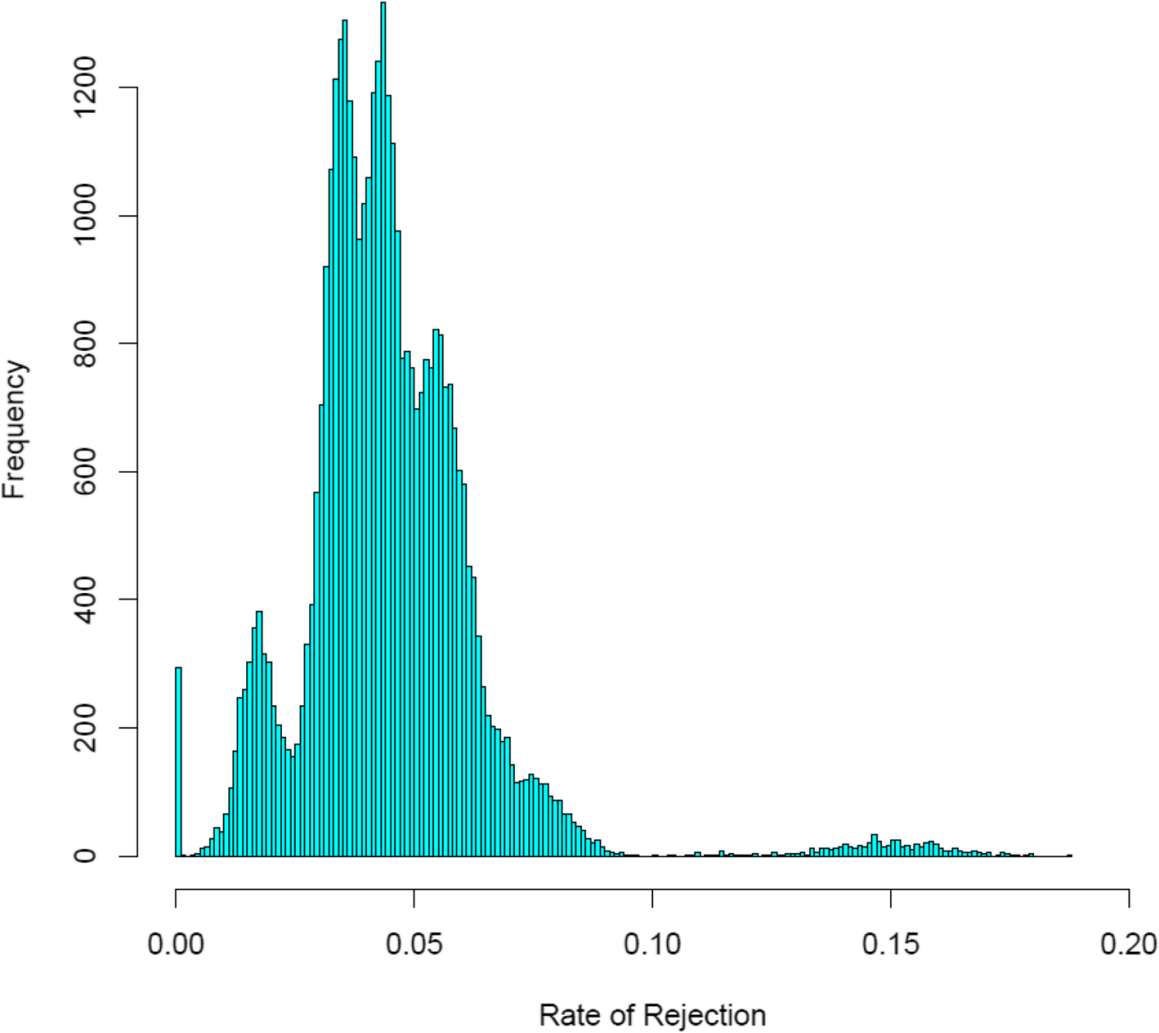
Distribution of the rate of rejection for all relationships in a crossbred experiment

In our dataset of Duroc × Iberian crossbreds, no relationship resulted in *τ*_*ij*_ = 0. Since we were aware that the dataset included some true dams and sires together with their offspring, these results indicate that all true first-degree relationships were rejected because of genotyping errors; indeed, there were relationships with *τ*_*ij*_ ≈ 0. The distribution of the rate of rejection of first-degree relationships τ_ij_, corresponding to all individuals and each relationship, in all relationship groups, is given in Fig. 1. Because several peaks can be distinguished, this figure suggests mixture of different underlying distributions corresponding to the distribution of the rate of rejection due to genotype errors *τ*_*g*_, and to the distribution of *τ*_*ij*_ corresponding to true rejection of first-degree relationships *τ*_*r*_, in the different relationship groups (Fig. 1).

**Fig. 2:**
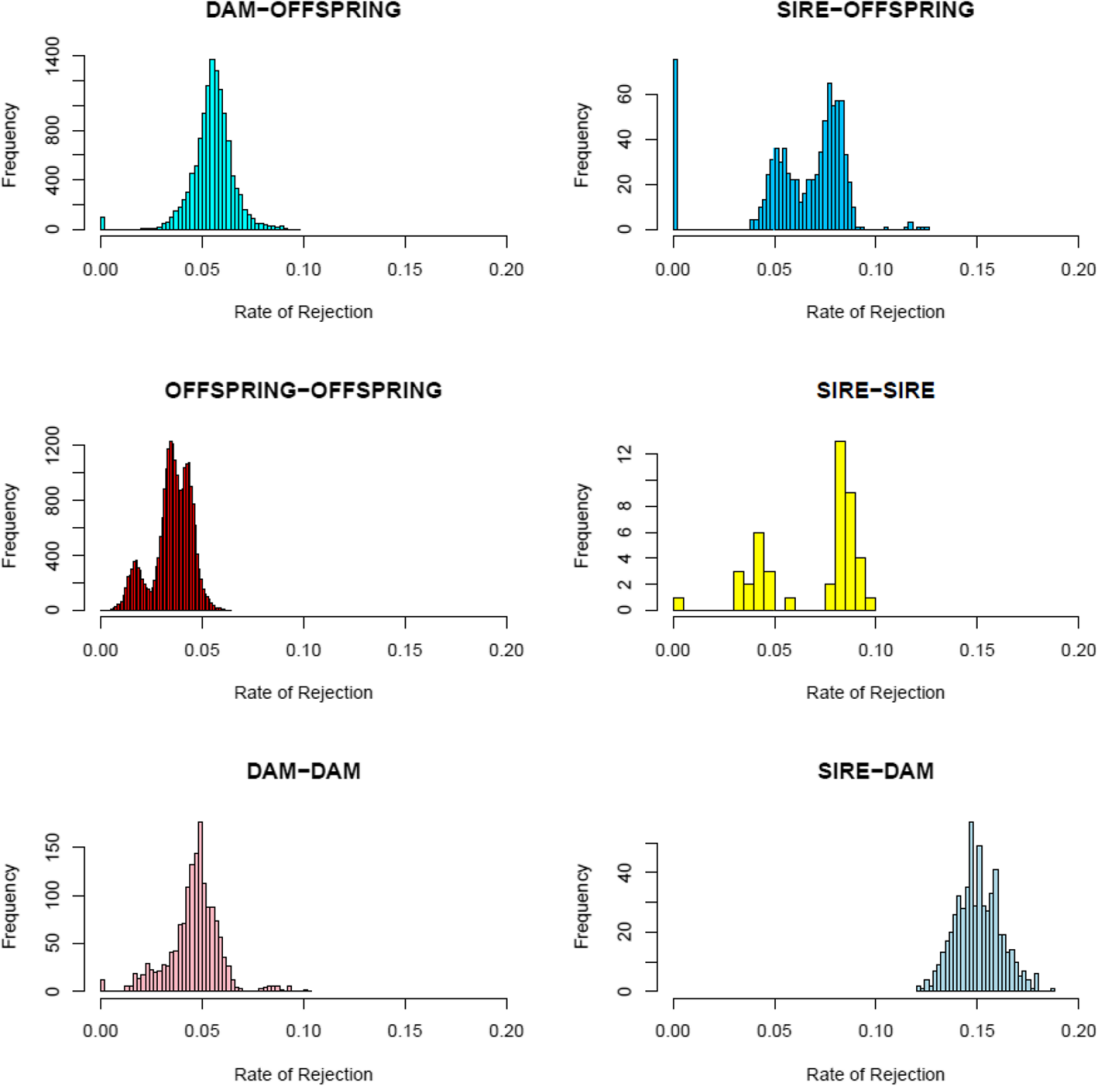
Distribution of the rate of rejection of first-degree relationships within dam-offspring, sire-offspring, offspring-offspring, sire-sire, dam-dam, and sire-dam relationship groups in a crossbred experiment

The distinction between the distributions of *τ*_*g*_ and *τ*_*r*_ becomes clearer when data is separated by relationship group: dam-offspring, sire-offspring, offspring-offspring, dam-dam, sire-sire, and sire-dam (Fig. 2). The distribution of *τ*_*ij*_ within the dam-offspring and within the sire-offspring groups shows a clear separation between rates of rejection of parent–offspring first-degree relationships *τ*_*ij*_ very close to zero (*τ*_*ij*_ ≈ 0), and between rates of rejection of first-degree relationships *τ*_*ij*_ with higher values (*τ*_*ij*_ > 0). In the parent–offspring groups, when the probability that two individuals are homozygous for alternative alleles at a given locus is very close to zero, their values correspond to genotyping errors (i.e., the distribution of *τ*_*g*_), while higher *τ*_*ij*_ values correspond to true rejection of first-degree relationships (i.e., the distribution of *τ*_*r*_). The distributions of *τ*_*g*_ and *τ*_*r*_ are more overlapping in the offspring-offspring group. This is expected from the observation that true full-sibs can be homozygous for alternative alleles when both parents are heterozygous, albeit at a low probability of 0.125. A clear distinction between values of τ_ij_ very close to zero and those farther away from zero can also be seen in the distribution of *τ*_*ij*_ in the dam-dam and sire-sire groups (Fig. 2). These values indicate the presence of one first-degree relationship in the sire-sire group and a number of first-degree relationships in the dam-dam group. In further analyses, it appeared that some samples were repeated; this may explain the observed sire-sire and dam-dam first-degree relationships. In the sire-dam group, no first degree relationship are detected, which is expected in parents from a cross-breeding experiment since dams and sires belong to two different breeds. The rate of rejection is very large showing the differences in genetics between the breeds.

### Expectation–Maximization to establish first-degree relationships

**Fig. 3:**
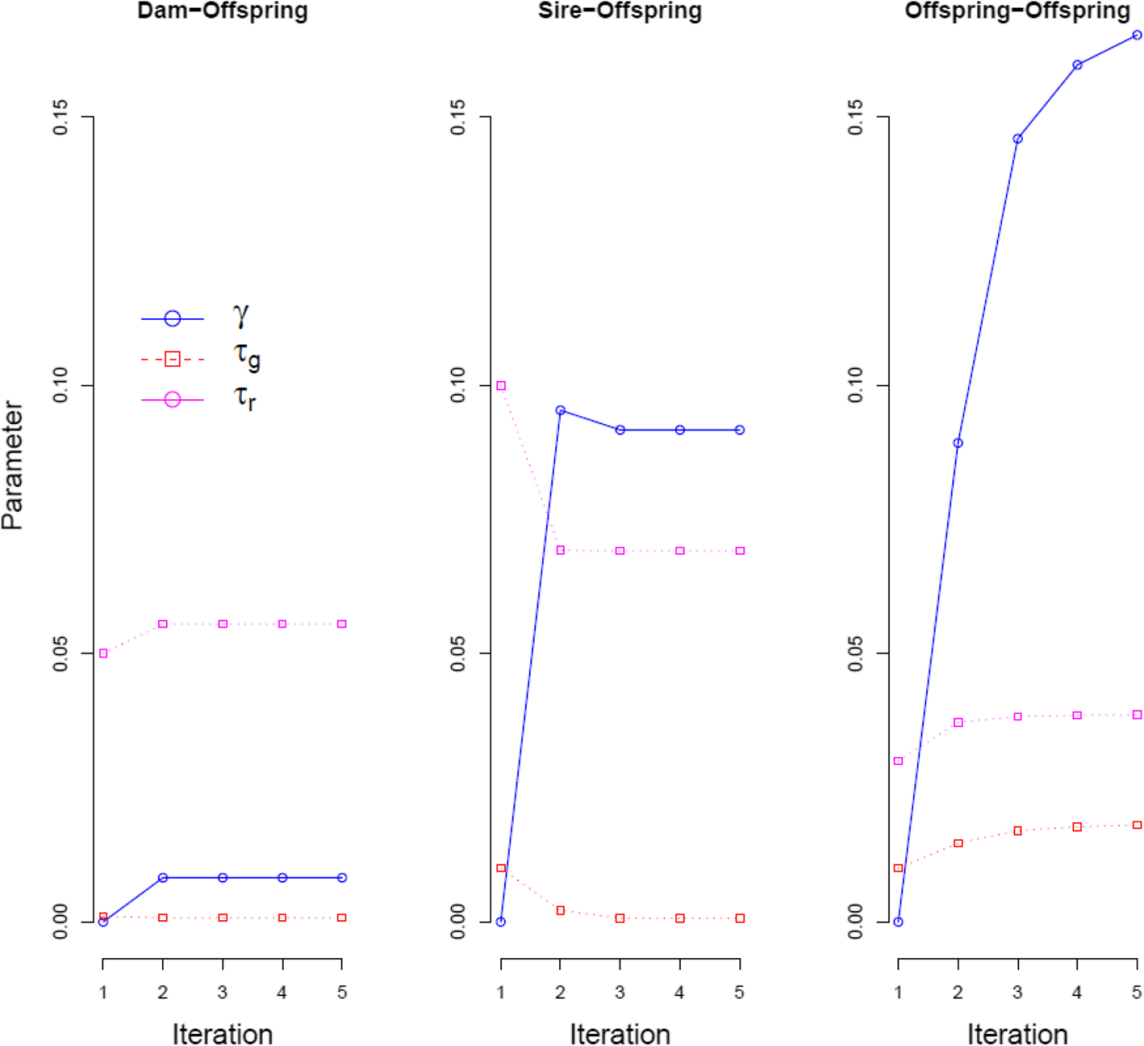
Convergence of the Expectation–Maximization algorithm to estimate first-degree relationships using Dam-Offspring, Sire-Offspring, and Offspring-Offspring relationships

Because due to the existence of genotyping errors *τ*_*ij*_ = 0 cannot be used as the only criterion on which to accept first-degree relationships, an Expectation–Maximization method was developed to establish whether values of *τ*_*ij*_ belong either to distribution *τ*_*g*_ (*τ*_*ij*_ ≈ 0; genotyping errors) or to distribution *τ*_*r*_ (*τ*_*ij*_ > 0; true rejection of first-degree relationships). Relationships with values of *τ*_*ij*_ that belong to distribution *τ*_*g*_ are true first-degree relationships. Figure 3 illustrates the convergence of the E-M algorithm for dam-offspring, sire-offspring, and offpring-offspring relationships. Convergence for all parameters took place in just two to three iterations. Initial values for *τ*_*g*_ and *τ*_*r*_ were chosen such that they were close to the peak of the corresponding distributions in Fig. 2. When the chosen initial values for *τ*_*g*_ and *τ*_*r*_ were not close to the corresponding peak of the distribution, parameters were not converging.

Applying the Expectation–Maximization algorithm, we established paternity of 75 and maternity of 99 piglets. We identified that 14 offspring out of 99 appeared to have two mothers. We could establish that the two mothers were the same individual, and blood for DNA extraction had been sent twice and with different identification to our lab. Therefore, we established 85 dam-offspring relationships. The reason why only 75 and 85 piglets were associated to sire or dam candidates, respectively, was that DNA of the true parents was not included in the dataset for all offspring, since those DNA samples had not been supplied by the farm. In addition, we established 3,802 full-sib relationships among the offspring-offspring relationship group. This figure is too large which indicates that the method cannot separate properly full-sibs from half-sibs singularly in a pig breeding farm, when sires or dams are close relatives (e.g., different sires in a herd can be brothers, or different dams in a herd can be sisters). This is illustrated by the overlap in the histogram of the offspring-offspring group in Fig. 2.

In summary, we analyzed samples of 288 individuals. Genotyping of one of the samples did not work, and six samples failed the threshold set for the calling rate. Statistical analysis detected 14 duplicated samples of dams. Of 9 candidate sires and 55 candidate dams, 7 sires and 38 dams were parents of the offspring. The sire with the largest number of offspring had 22 offspring; the dam with the largest number of offspring had 6 offspring. There were 27 piglets with paternity and maternity simultaneously identified.

### Estimation of genotyping errors

After establishing relationships with τ_ij_ ≈ 0 that belong to distribution τ_g_, we established that the genotyping error estimated jointly in sire-offspring and dam-offspring relationship groups was 0.000735. This is a lower bound of the true number of genotyping errors since genotyping errors are only identified for SNP markers in parent–offspring relationships for which individuals are homozygous for alternative alleles; other genotyping errors involving heterozygous animals in either parent or offspring remain undetected. The true genotyping error depends on the allele frequency of the marker. For markers with a very low allele frequency, the rate of rejection approaches the genotyping error. In this situation, true rejection is difficult to occur because the homozygote corresponding to the allele with very low frequency is very scarce; therefore, rejection can only be attributed to genotyping error. In our experiment, the genotyping error is estimated at 0.006 (0.6%).

### Detection of SNPs with a high rate of rejection of first-degree relationships in true parent–offspring relationships

**Fig. 4:**
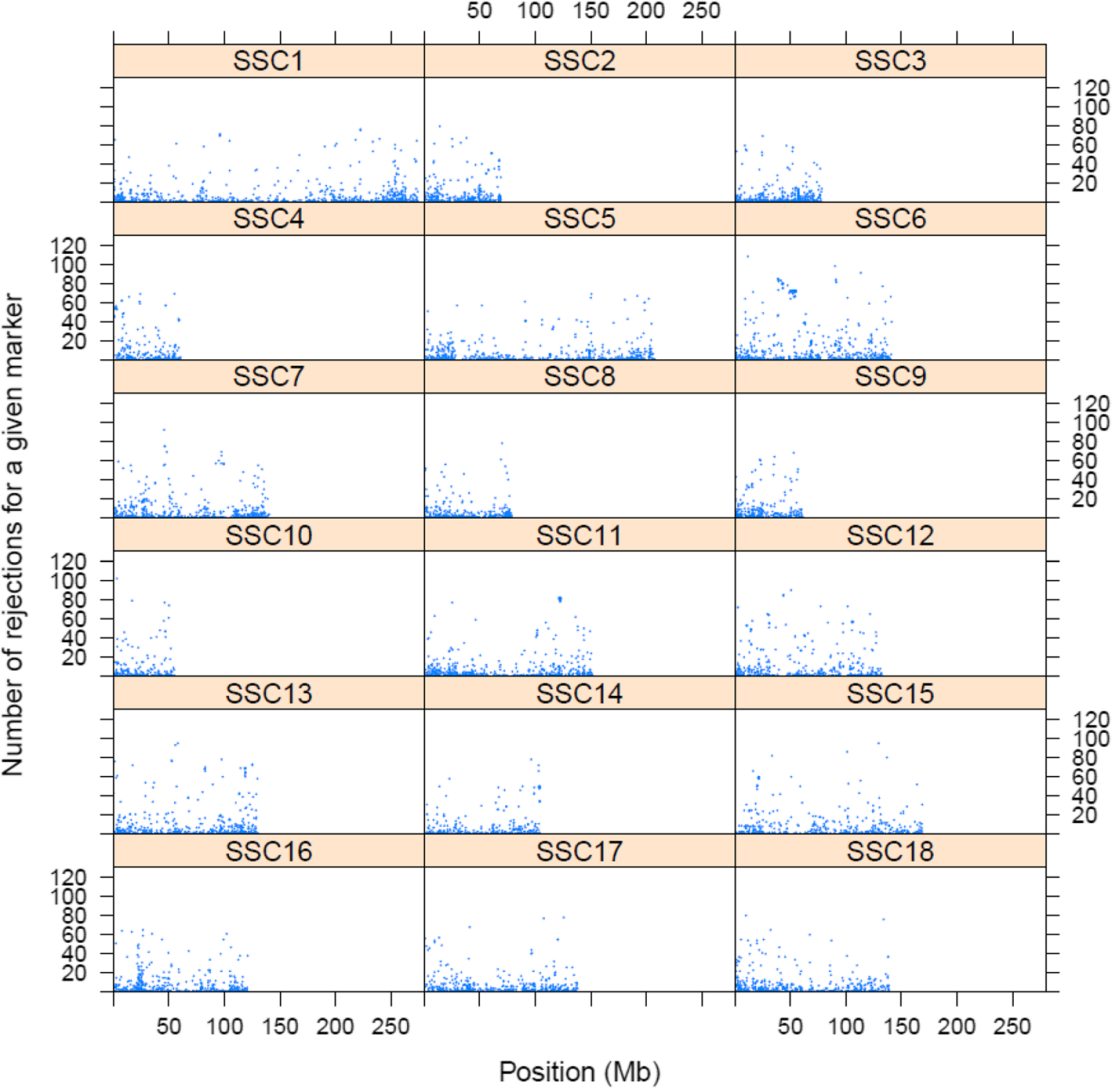
Genome-wide number of rejections for SNPs rejecting true parent–offspring relationships

**Fig. 5:**
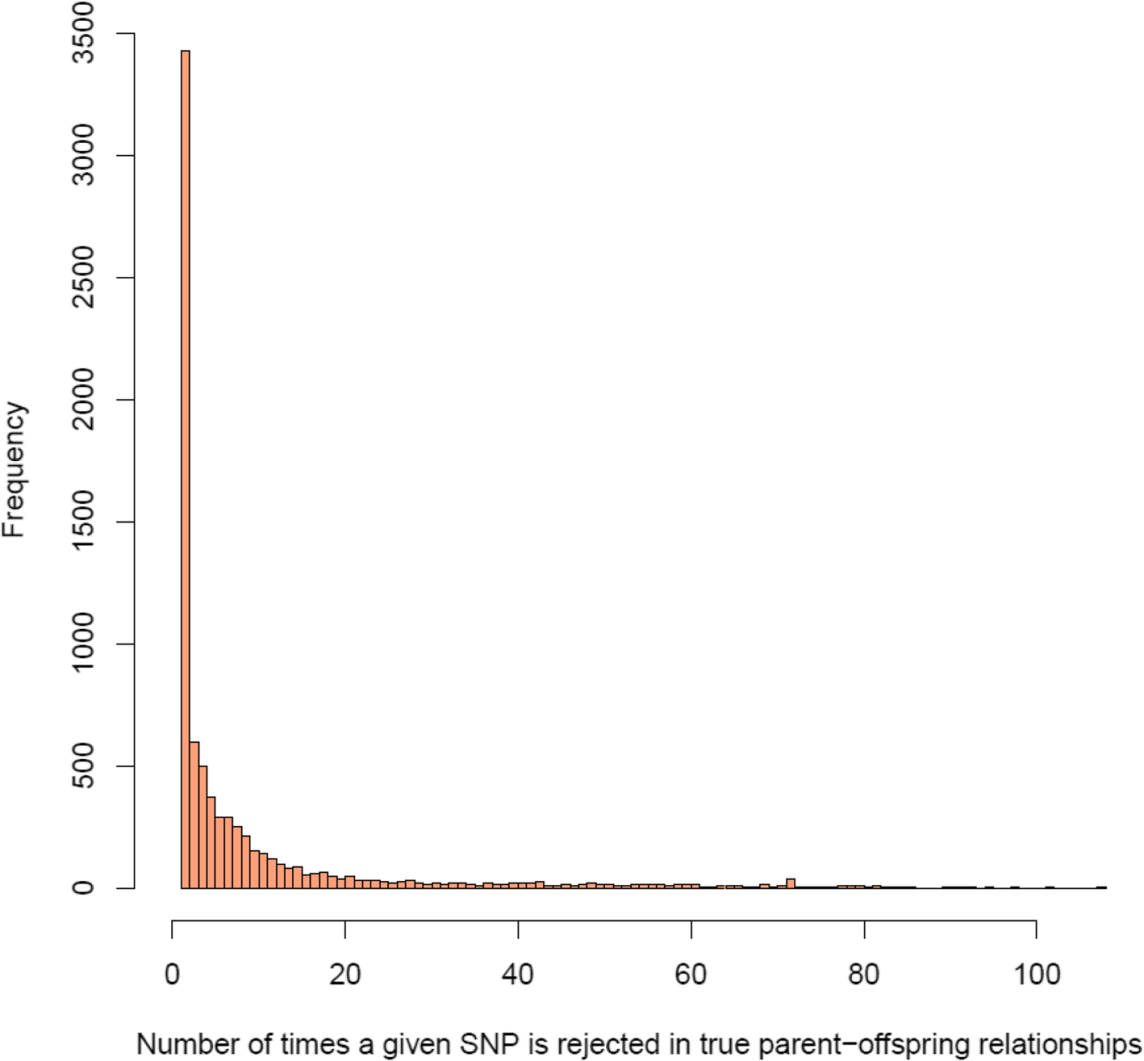
Histogram of the number of times a given SNP is rejected true parent–offspring relationships

**Fig. 6:**
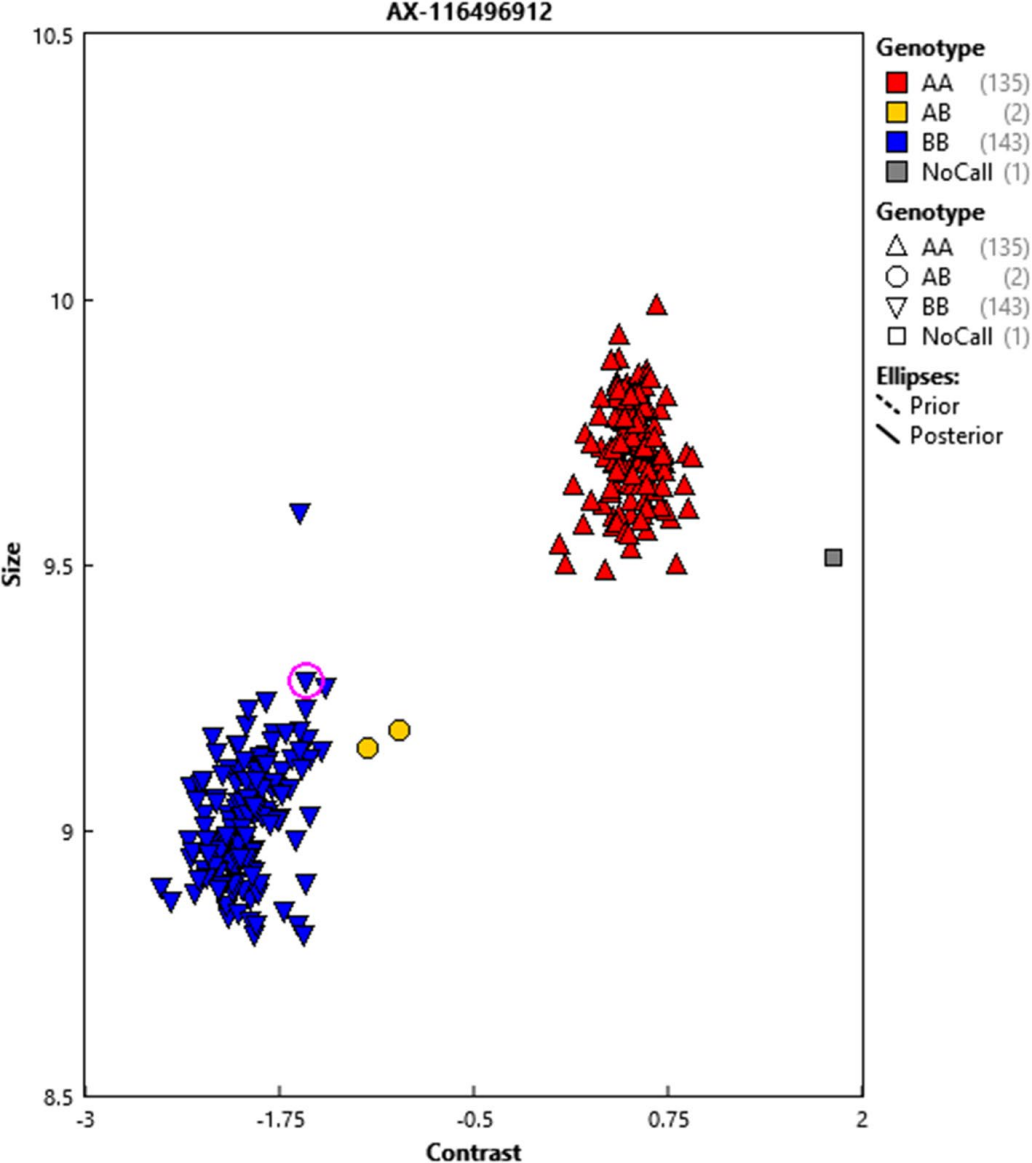
Cluster for SNP Affx-115138382 (AX-116496912) that rejected 108 times true first-degree relationships

In our dataset, a total of 69,882 rejections of true relationships corresponding to 7744 SNP markers were observed in the parent–offspring groups. The genome-wide distribution of those SNPs is given in Fig. 4. We can now identify SNPs that repeatedly rejected first-degree relationships in true parent–offspring pairs. The majority of the 7744 SNPs rejected first-degree relationships in true parent–offspring only a few times, however, some of the SNPs rejected first-degree relationships particularly often (Fig. 5). We identified 3,224 SNPs rejecting five or more true parent–offspring relationships (Fig. 5); these SNPs were considered ‘failing’ and are reported in additional file 2. For example, SNP with Affymetrix identification Affx-115138382 (AX-116496912) and mapped to position 11,597,555 on SSC14 rejected 108 true parent–offspring relationships. Fig. 6 shows the cluster provided by the Axiom suite analysis of the genotypes of all parents and offspring for this SNP; only two individuals are heterozygous. This is an indication that this SNP does not follow autosomal Mendelian rules of inheritance; since the two animals are crossbreds, the observed homozygous genotypes could only come about when both their sire and dam are homozygous for the same allele. These results could be attributed to genotyping errors, but also to wrong (non-autosomal) mapping locations, structural variations, etc.

For the analyses performed in the present study, SNPs were not filtered for departure of Hardy–Weinberg equilibrium (HW) and/or Minimum Allele Frequency (MAF). Since the offspring are a result of a cross between Iberian × Duroc, it is expected that SNPs are not necessarily in HW equilibrium. There were 122,615 autosomal and mapped SNPs with MAF < 0.05 in the total data set, corresponding to a proportion of 0.224 (122,615/546,220). Only 165 out of a total of 7,744 SNPs that rejected true parent–offspring relationships had a MAF < 0.05, corresponding to a proportion of 0.021 (165/7,744). As aforementioned, there is a reduced capacity of markers with low allele frequency to reject true parent–offspring relationships because the chances of being heterozygous for alternative alleles is very low (one of the two homozygotes is at very low frequency). SNPs at intermediate frequencies are more likely to reject first-degree relationships.

## Discussion

The present study shows the successful application of an Expectation–Maximization (EM) algorithm to establish or to verify first-degree parent–offspring relationships when relationships are unknown or uncertain, in an animal population that is genotyped with a High-Density (HD) SNP Array. This method has a wide range of applications. In livestock populations, first-degree relationships are nearly always present. Although the advent of genomic methods meant that pedigree information necessary for breeding value estimation can now be replaced by genomic relationships in genomic best linear unbiased prediction (GBLUP) [10], and the inbreeding coefficient of individuals necessary to control economic losses from inbreeding depression [11] can be estimated from molecular marker data [12], accurate pedigree information is still key to animal populations where molecular information is not routinely available (e.g., [13]). However, collecting accurate pedigree information may be demanding because of gaps in data recording, loss of records, inadvertent errors in animal labelling or registration, inability to cost-effectively identify animals individually, or the inability to assign parents to offspring. For example, individual identification of small fish is often demanding, such that fish are commonly kept in families until they are large enough to be individually tagged [14]. Assigning parents to offspring is not straightforward in multi-sire breeding schemes, such as those employed in cattle ranching operations where breeding and calving is unassisted and, therefore, it is not possible to correctly assign paternity (and sometimes maternity) to a given calf [15]. Paternity and maternity identification errors may substantially negatively impact estimated breeding values, genetic trends, inbreeding, and result in the inability to identify truly superior animals in a population [16]. Furthermore, parentage identification is important to companion animals to evaluate inbreeding and genetic diversity, pedigree structure, and for registration purposes [17], to free-living animal populations to evaluate population and kinship structure and genetic diversity [18], and for forensic human identification [19].

While the first parentage verification methods used blood groups [20], this was replaced by an international standard of the International Society of Animal Genetics (ISAG) for parentage verification and identity testing following DNA typing based on microsatellite markers [21], and more recently also based on SNP markers [22]. Microsatellites are polymorphic codominant genetic markers containing repeated nucleotide sequences, with 2–10 nucleotides per repeated unit, that are present across the genome. Although they have been used and are still used widely for parentage verification and identity testing, some problems persist: microsatellite markers are not highly polymorphic in all species, the scoring of markers is not straightforward or automated, and they require a rather large (initial) investment in terms of labor and financial resources [23]. These problems were largely overcome by the development of SNP markers which have only just been successfully applied in parentage verification in livestock [24], fish [25], companion animals [17], wild animals [18], and humans [19].

Because microsatellites are more polymorphic than the bi-allelic SNPs, parentage verification is accomplished by a larger number of SNPs than microsatellites in a panel. Between approximately 40 and 100 SNPs are equivalent to between 14 and 20 microsatellites; ISAG recommends a minimum number of 100 SNPs for parentage testing [26]. Microsatellite and SNP panels for parentage verification are successful and particularly useful when HD SNP panels are too expensive for routine use, e.g., in smallholder systems [27]. However, the Expectation–Maximization (EM) method applied to all SNPs in an HD SNP Array described in the present study has several additional advantages in populations where HD SNP Arrays are routinely applied, e.g., for genome-wide association studies (GWAS) and/or genomic selection, or when there are no cost limitations. Firstly, whereas microsatellite and SNP panels for parentage identification are designed and verified in different species and made available internationally (e.g., the Bovine ISAG SNP Parentage Panel based on 200 bovine SNP markers selected by ISAG) or may be tailored to specific breeds, the EM method can be applied to HD genotyped animals without prior evaluation and verification. Secondly, parentage test panels do not take into account genotyping errors. The EM method is easily applied to evaluate (a lower bound of) genotyping errors in individual experimental datasets, by identifying true parent–offspring that are homozygous for alternative alleles. This method can be applied to investigate genotyping errors in different datasets using the same array. In addition, the nature of genotyping errors can be further investigated. For example, we identified “problematic” SNPs that particularly often rejected true first-degree relationships. Those should be eliminated from the data set for GWAS or Genomic Selection, but they should also be further investigated in order to understand why they do not follow autosomal Mendelian inheritance rules.

The method described in this study can be easily applied to animal populations for which a SNP array is available for the species in question. Although the present study was performed with an HD SNP array, arrays at lower densities are expected to be capable to draw similar conclusions and will be cheaper. Routine application of genomic selection requires at least a low-density array. Verification or detection of first-degree relationships, together with a measure of the genotyping errors of each batch, may facilitate detection of errors and assure the quality of genotyping. The required number of SNPs needed to detect if any given relationship in a group of individuals belongs to either *τ*_*g*_ or *τ*_*r*_ can be approximated by computing the statistical power using the normal approximation to the binomial distribution (https://www.stat.ubc.ca/~rollin/stats/ssize/b1.html). For example, for a group of 300 individuals in which no assumptions can be made about candidate parents or offspring, the number of combinations (tests) of all possible relationship pairs is 300 × 299 / 2 = 44,850. This figure is needed for adjusting the significance level to multiple testing. Thus, a significance level of 0.01 is adjusted to 0.01/44,850 using the Bonferroni adjustment. For a statistical power of 0.99 at a global significance level of 0.01 with *τ*_*g*_ = 0.000735 and *τ*_*r*_ = 0.01, the number of SNPs required is 1,611 for 300 individuals. Therefore, detection of parent–offspring relationships should be possible with arrays of a low, medium or a high density unless the number of individuals is very high.

A simple statistic proposed in this study, rate of rejection of first-degree relationships, is helpful to verify or to detect paternities and maternities when testing a large number of SNPs. It is also shown that the rate of rejection allows estimation of different types of relationships, and even genetic differences between groups of animals when sires and dams belong to different breeds. This statistic is based on the simple exclusion rule that two individuals that are homozygous for alternative alleles cannot have a parent–offspring relationship. A more complete assessment including likelihoods of all possible first, second and third-degree relationships has been proposed by Huisman [10]. That work is aimed at reconstructing multigenerational pedigrees with a reduced number of SNPs. However, the use of Huisman's [10] approach with full arrays either at high or low density and a large number of animals is computationally impractical with today’s computer capacities. In addition, our approach based on many thousands of SNPs allows accurate estimation of genotyping errors and identification of problematic SNPs wrongly rejecting many true parent–offspring relationships.

## Conclusions

SNP arrays can be used to test or to verify paternities using the rate of rejection of first-degree relationships. The same material can be used to estimate genotyping errors due to the large number of SNPs tested in parent–offspring relationships. This approach helps to identify SNPs inconsistent with Mendelian rules of inheritance in multiple parent–offspring relationships.

## Supplementary Information

**Additional file 1.** 

**Additional file 2.**

## Data Availability

The genotype files generated and/or analyzed for the current study are available at Zenodo with https://doi.org/10.5281/zenodo.5255900.
