# Supplementary material for: First-degree relationships and genotyping errors deciphered by a high-density SNP array in a Duroc × Iberian pig cross
Source: BMC Genom Data. 2022 Feb 17;23:14. doi: 10.1186/s12863-022-01025-1 (PMC8851823; doi:10.1186/s12863-022-01025-1)
Supplement: Supplementary file 1 — Additional file 1. [file 12863_2022_1025_MOESM1_ESM.zip › INFORMATION-PROGRAMS.pdf]

**Programs in R to estimate rate of rejection, paternities and genotyping errors**

There are four different programs that need to be executed consecutively. The programs in R store all information in the memory and you may require a large amount of memory in your computer in order to run all programs. Only autosomal SNPs mapped to SScroffa v11.1 are used.

1) The program chro.r reads input file "iber.TPED" exported from the Axiom Suite software of Affymetrix and available at Zenodo with DOI 10.5281/zenodo.5255900. This file has four rows corresponding to the markers and four columns corresponding to the individuals. It computes all relationships from all individuals and optionally writes it to the file "rejection", which holds all relationships and markers rejected for first-degree relationships. This is a very time consuming process. This writing part is optional, and to run it you just need to remove the # at the end of the program (in bold). A matrix, rel, is created with all numbers of rejections from any pair of individuals in the data set. The program is slow (comparisons in R are very slow). It takes 18 hours for a PC with 64 RAM without the writing part. With a smaller data set, the running time of the program should be considerably faster.

2) The program rel.r uses the square matrix of all possible relationships generated in the previous program to create a data frame with 7 columns: ind1 (individual 1), ind2 (individual 2), re (number of SNPs rejecting that relationship), rela1 (kind of first individual: sire, dam, or offspring), rela2 (kind of second individual: sire, dam, or offspring), r1 (number of order of first individual), r2 (number of order of second individual). The rate of rejection is the value of "re" divided by the number of markers. This program also separates the relationship after type of relationship group: sire-offspring, dam-offspring, offspring-offspring, dam-dam, sire-sire, and dam-sire. In addition, the program plots histograms of the relationships. The program requires a list of all individuals as is given for this dataset with name "names".

3) The program re123.r computes the Expectation-Maximization of the dam-offspring, sire-offspring, and offspring-relationships. It produces data frames with all true sire-offspring, dam-offspring, and offspring-offspring first-degree relationships with names sires, dams, fullsib, respectively.

4) The program gerror.r merges all true sire-offspring and dam-offspring relationships in files 'dams' and 'sires' (this is obtained by Expectation Maximization just like with program re123.r). The program also plots a lattice with the distribution of the number of SNPs rejecting first-degree relationships by chromosomal position. In addition, a list of the SNPs rejecting six or more true parent offspring relationships is written to file SUPLEMENTAL MATERIAL 2.
